# A meta-analysis of selected near-road air pollutants based on concentration decay rates

**DOI:** 10.1016/j.heliyon.2019.e02236

**Published:** 2019-08-23

**Authors:** Shi V. Liu, Fu-lin Chen, Jianping Xue

**Affiliations:** National Exposure Research Laboratory, Office of Research and Development, U. S. Environmental Protection Agency, RTP, NC, 27711, USA

**Keywords:** Environmental sciences, Atmospheric science, Near-road, Concentration decay, Meta-data analysis, Traffic, Air pollution, Distance, Vehicle emission

## Abstract

Traffic-related air pollution has been associated with various health risks for human populations living near roadways. Understanding the relationship between traffic density and dispersion of vehicle-released air pollutants is important for assessing human exposure to near-road air pollutants. We performed a literature survey targeting publications containing measurement data of traffic-related air pollutants near roads with distance information on their concentration distribution. Concentration decay rates over down-wind distance away from major roads were calculated for black carbon (BC), carbon monoxide (CO) and nitrogen oxides (NO_2_ or NO_x_) and meta-data analysis on these rates was performed. These analyses showed metadata-based exponential decay rates of 0.0026, 0.0019, 0.0004, and 0.0027 m^−1^ for BC, CO, NO_2_ and NOx, respectively. Using these measurement data-based decay rates, concentrations for BC, CO, NO_2_ and NO_x_ over various near-road distances were predicted. These results are useful for enhancing exposure modeling and thus more reliably assessing the health risk of exposure to near road air pollution.

## 1 Introduction

Petroleum-fueled motor vehicles release regulated air pollutants such as black carbon (BC), carbon monoxide (CO), nitric oxides (NO_x_), and particulate matter (PM) [1, 2, 3, 4, 5]. The health impact of these mobile source air pollutants on human populations has been reflected with increasing observations on elevated adverse health effects among near-road residents [6, 7, 8, 9, 10, 11, 12]. With an increasing trend of urbanization in human societies and a growing dependency on motor vehicles for economic functions, traffic-related air pollution is becoming more and more significant [13, 14].

Compared with point-source air pollution associated with a fixed location, mobile source air pollution is more difficult to evaluate. Many studies have been carried out to characterize the spatial distribution of mobile source air pollutants [15, 16, 17]. Most of these studies report monitoring data for selected air pollutants at a specific location [18]. These studies are valuable for site-specific discussion of potential exposure of local residents to the studied air pollutants. Unfortunately, such extensive studies are simply too expensive to be carried out in many communities where near-road air pollution is prominent.

There is an urgent need for developing some generalized knowledge on common spatial patterns of vehicle-emitted pollutants [19, 20, 21]. These general understandings for near-road air pollution patterns are useful in many ways [22, 23]. For example, they can be used for assisting policy-making especially in areas lacking monitoring data [24]. They can also be used for evaluating development-planning so that traffic pollution can be minimized if not eliminated [25].

Such generalization can be achieved through meta-data analysis on large data sets consolidated from multiple studies. A meta-analysis has tried to evaluate factors influencing the spatial extent of mobile source air pollution [26]. It showed a complex interplay of spatial extent definitions, emission rates, background concentrations, and meteorological conditions on spatial extent estimates even for non-reactive pollutants. It calls for “high resolution” modeling near the source and developing a “zone of influence” for mobile sources.

We performed a new meta-data analysis in an aim to address the needs identified in the previous meta-data analysis and, with a more comprehensive coverage of different studies and a more detailed data analysis, intending to reach some refined understanding of spatial distribution of traffic-related air pollutants. We found that, by using pollutant concentration decay rate as a common parameter for evaluation, some spatial profiles can be seen more clearly for traffic-related air pollutants near roads. The concentration decay rate-based analysis will provide some detailed knowledge for more confident estimates on near-road spatial distribution of traffic-related air pollutants. This knowledge will be very important for evaluating exposure levels of human populations living at various distances to the mobile source of air pollutants [24, 25].

## 2 Materials and methods

### 2.1 Literature search

Using ProQuest we searched its Environmental Science Collection databases for broad scope terms that might hit publications containing information on distance distribution of air pollutants near roadways (see Table 1 for search criteria). Because our goal was to get publications containing concentration measurement data on near-road air pollutants we searched the initial matches again to exclude those reporting models, health effects or land uses. From those matched findings we then focused on a selected set of air pollutants which included BC, CO, NO_2_, NO_x_, and PM (PM_2.5_ and PM_10_). Publications matching these selections were exported into an EndNote library. Then titles of these publications were manually screened by three researchers independently to identify genuine matches. The results of this screening were combined and the abstracts of each paper were again independently read by three researchers to reach the final set of papers containing measurement data on at least one of the selected air pollutants.

**Table 1:** Literature search criteria and results.

Step	Search criteria and procedures	Database searched and library created	Results
1	(air pollution measurement) AND (major road OR highway) AND (distance OR spatial distribution) in anywhere	ProQuest Environmental Science Collection Was searched using criteria specified.	6021
2	Above criteria plus “NOT *” where * is “model”, “health effect”, or “land use”	ProQuest Environmental Science Collection Was searched with different exclusion terms and then results are combined into an EndNote library.	3799
3	Endnote library was searched for specific air pollutants such as “black carbon”, “carbon monoxide”, “nitrogen oxides”, or “particulate”	Groups containing publication matching each specific air pollutant were created within Endnote library.	1879
4	Title reading by three researchers independently	Publications passing concurrent agreement of three researchers' screening were collected into a separate group within the EndNote library.	373
5	Independent reading of abstracts by three researchers	Publications containing useful measurement data on selected air pollutant were collected into final concurrence set.	65

### 2.2 Data extraction and collection

A master spreadsheet was created to collect pollutant concentrations over distances from roads. Original numerical data contained in a publication or obtained from authors were used when they were available but most times estimated values obtained from published figures were used. Because initial inspection on the extracted data showed no obvious concentration decay over distance away from road for PM which did not include ultrafine PM we limited our continued data compilation only to BC, CO, NO_2_, and NOx. In addition to extracting data from publications found in the literature search we also obtained original data from large EPA-sponsored measurement studies such as the Las Vegas study [27] and the near-road exposures and effects of urban air pollutants study (NEXUS) [28].

### 2.3 Data analysis

Pollutant concentration decay rate (R) was calculated according to the following formula:

R = Ln (Ca/Ci)/D, where R is the decay rate, D is the distance between pollutant's concentrations at the initial measurement site (Ci) and after a distance (Ca). Ln represents natural logarithm.

The above two EPA-sponsored near-road air pollutant measurement studies were analyzed first using SAS program to see concentration distribution pattern of BC and to obtain means and standard deviations of air pollutant concentrations over monitoring distances. Pollutants’ decay rates were calculated for distance range 20–100 and 100–300 m as well as 20–300 m.

Meta-data analysis on calculated concentration decay rates was performed according to random model described [29]. Specifically, a random effects model was used for the meta-analyses. In the Random effects model, the true effect could vary from study to study while one true effect size was assumed in the fixed-effect model. Mean, standard error and sample size were used for meta-analyses to calculate the overall decay rate across all studies. Missing values of standard deviation for decay rates in literature-extracted studies were filled in with equivalent values derived from analysis on two large data sets of original measurement data from the above two EPA-sponsored studies.

## 3 Results

### 3.1 Scientific interest and research depth on near road air pollution

With our initial search criteria we found 6021 publications in the broad search performed in July 2013 (Table 1). However, as our focus in this study was to better understand the spatial extent or dynamics of near-road air pollutants, publications dealing with models, health effects, or land use were excluded from the initial matches and only 3799 publications were retained. With a further limitation of our focus on BC, CO, NO_2_, NOx, and PM we narrowed our collection to 1879 publications which were imported into an Endnote library. Three researchers independently reviewed the titles of this collection to make their respective selections. Then a total of 373 publications were collected after discussion. The abstracts of this collected set were independently read by three researchers and a final set of 65 publications were found to contain measurement data on at least one of the selected air pollutants with a kind of distance profile from major road.

### 3.2 Distance and concentration ranges of selected air pollutant in various studies

Initial evaluation of those 65 publications indicated that PMs showed no apparent concentration decay over within near-road distances. Thus we focused our spatial profile analysis of concentration decay only on BC, CO, NO_2_ and NO_x._ We found 12 unique studies contained sufficient concentration and distance measurement information for our concentration decay analysis (Table 2).

**Table 2:** Near-road distance-concentration profiles of selected air pollutants.

Pollutant	Study	Distance Range, m	Concentration Range	Note	Ref.
BC	26	20–300	1.52–0.78	All direction	[17]
BC	26	20–300	1.68–0.82	Downwind	[17]
BC	35	5–450	3.5–1.8	upwind	[30]
BC	35	5–500	5–2	Downwind	[30]
BC	48	0–60	9–1.4	Downwind	[31]
BC	55	15–305	14.9–7.4	Location Delft, Downwind	[32]
BC	55	32–260	12.2–8.7	Location Overschie, Downwind	[32]
BC	65	35–300	5.5–1	405 Freeway, Downwind	[33]
BC	65	15–300	22–5	710 freeway, Downwind	[33]
BC	65	17–300	21.7–5.5	Both freeway, Downwind	[33]
CO	10	10–1450	195–30	SH – 71, Parallel	[34]
CO	10	150–390	250–130	I – 35, Parallel	[34]
CO	10	250–1750	75–60	FM – 973, Parallel, Upwind	[34]
CO	10	5–1100	190–60	FM –973, Parallel, Downwind	[34]
CO	10	20–25	150–140	I – 35, Perpendicular, Downwind	[34]
CO	10	75–175	75–45	FM – 973, Perpendicular, Upwind	[34]
CO	10	5–220	125–60	FM – 973, Perpendicular, Downwind	[34]
CO	20	20–150	210–180	S1 – S2, Upwind	[16]
CO	20	40–150	400–320	N1 – N4, Downwind	[16]
CO	26	20–300	340–270	All direction	[17]
CO	26	20–300	400–300	West	[17]
CO	44	25–1000	500–300	SW, Upwind	[35]
CO	44	25–325	400–300	NE, Downwind	[35]
CO	49	9–53	850–500	Upwind	[36]
CO	49	0–80	1050–460	Downwind	[36]
CO	65	35–300	2000–300	405 Freeway, Downwind	[33]
CO	65	15–300	2300–100	710 Freeway, Downwind	[33]
CO	65	17–300	2300–200	Both Freeway, Downwind	[33]
NO_2_	10	10–1500	5.5–1.5	SH – 71, Parallel	[34]
NO_2_	10	100–400	10.5–3	I – 35, Parallel	[34]
NO_2_	10	100–1000	3.5–2	FM – 973, Parallel, Upwind	[34]
NO_2_	10	10–1100	9–2	FM – 973, Parallel, Downwind	[34]
NO_2_	10	20–25	13.5–13	I – 35, Perpendicular	[34]
NO_2_	10	90–150	2–2	FM – 973, Perpendicular, Upwind	[34]
NO_2_	10	10–220	8–3	FM – 973, Perpendicular, Downwind	[34]
NO_2_	14	50–200	20–13	Upwind	[34]
NO_2_	14	50–400	30–18	Downwind	[34]
NO_2_	15	0–950	32–12	Upwind	[20]
NO_2_	15	0–1300	32–15	Downwind	[20]
NO_2_	26	20–300	24.1–18.69	All direction	[17]
NO_2_	26	20–300	27.31–20.95	West	[17]
NO_2_	29	4–48	30–15		[38]
NO_2_	35	5–450	30–25	Upwind	[30]
NO_2_	35	5–500	32–25	Downwind	[30]
NO_2_	36	30–60	5.4–4.5	Location A	[39]
NO_2_	36	10–30	5.8–5.6	Location B	[39]
NO_2_	36	10–30	6.9–6.6	Location C	[39]
NO_2_	48	0–60	70.5–59.2	Downwind	[31]
NO_2_	55	15–305	47.8–30.6	Location Delft, Downwind	[32]
NO_2_	55	32–260	44.8–32.1	Location Overschie, Downwind	[32]
NO_x_	10	10–1500	17–3	SH – 71, Parallel	[34]
NO_x_	10	100–400	25–5	I – 35, Parallel	[34]
NO_x_	10	100–1000	7–3	FM – 973, Parallel, Upwind	[34]
NO_x_	10	10–1100	32–3	FM – 973, Parallel, Downwind	[34]
NO_x_	10	20–25	38–33	I -35, Perpendicular, Downwind	[34]
NO_x_	10	90–150	4–3	FM – 973, Perpendicular, Upwind	[34]
NO_x_	10	10–220	40–5	FM – 973, Perpendicular, Downwind	[34]
NO_x_	14	50–200	35–25	I – 93, Upwind	[37]
NO_x_	14	50–400	65–30	I -93, Downwind	[37]
NO_x_	26	20–300	24.1–18.69	All direction, first period	[17]
NO_x_	26	20–300	27.31–20.95	West, first period	[17]
NO_x_	26	20–300	46.89–30.64	All direction, Second period	[17]
NO_x_	26	20–300	56.08–34.86	West, Second period	[17]
NO_x_	36	30–60	8.7–6.6	Location A	[39]
NO_x_	36	10–30	11.5–9.7	Location B	[39]
NO_x_	36	10–30	10.4–10.1	Location C	[39]
NO_x_	44	25–550	50–28	SouthWest	[35]
NO_x_	44	25–325	35–20	Northeast	[35]
NO_x_	48	0–60	189.9–93.6		[31]
NO_x_	49	0–53	200–80	Upwind	[36]
NO_x_	49	0–80	200–100	Downwind	[36]

Concentration as ug/m^3^ for BC and ppb for CO, NO_2_ and NOx, respectively.

Most of the collected studies showed measurement distance ranges up to 300 m while a few studies showed measurement distance up to 1500 m. In general, larger distance ranges were covered by traffic-related air pollutants in down-wind direction than those in up-wind direction. However, the actual concentration ranges varied greatly among different studies, indicating complex effects of local landscape, road construction, traffic density, and meteorology.

### 3.3 Decay rate of air pollutants in near-road distances

Initial inspection of a selected group of measurement-based studies revealed that traffic-related air pollutants often had a larger impact range in the down-wind than in the up-wind area. Thus, we selected only down-wind measurement data for calculating decay rates for a selected set of air pollutants. Based on our initial analysis on rich measurement data obtained from EPA-sponsored near-road air pollution study in Las Vegas, we found that concentration decay of BC was better described with two different rates for the 20–100- and the 100–300-meter distance ranges, respectively, rather than with a single rate covering the entire distance range. Thus, we used 20–100- and 100–300-meter distance ranges for separate decay rate calculations in addition to a whole-range decay rate calculation for all other studies (Table 3).

**Table 3:** Decay rates of air pollutants in different ranges of distance from road.

Pollutant	Study∗	Decay Rate (m^−1^)		
		20∼100 m	100∼300 m	20∼300 m
BC	LV	0.0028	0.0015	0.0018
	DX	0.0049	0.0013	0.0024
	O35	0.0023	0.0018	0.0024
	O48	0.0123	0.0061	0.0073
	O55	0.0048	0.0011	0.0024
	O65	0.0130	0.0017	0.0045
CO	LV	0.0012	0.0004	0.0009
	DX	0.0048	0.0002	0.0011
	O10-FM-973	0.0054	0.0002	-0.0034
	O10-SH-71	0.0069	0.0004	0.0050
	O44	0.0014	0.0001	0.0007
	O49	0.0120	NA	NA
	O65	0.0198	0.0044	0.0082
NO2	LV	0.0018	0.0008	0.0011
	DX	0.0020	-0.0004	0.0004
	O10	0.0064	-0.0013	0.0029
	O15	0.0041	0.0014	0.0021
	O26	0.0017	0.0006	0.0009
	O35	0.0014	-0.0003	0.0002
	O55	0.0039	0.0005	0.0011
NOx	LV	0.0035	0.0010	0.0018
	DX	0.0087	0.0002	0.0028
	O10	0.0225	0.0031	0.0064
	O26	0.0033	0.0027	0.0015
	O44	0.0029	0.0008	0.0023
	O49	0.0072	0.0037	NA

∗ Studies identified as “LV” and “DX” are for Las Vegas study [28] and the near-road exposures and effects of urban air pollutants study (NEXUS) [29], respectively. Other studies identified as “O##“, plus additional suffix to identify sub-sets of data within the same study (see Table 2 for details).

The mean decay rates for BC are always larger in distance range 20–100 m than in distance range 100–200 m while the whole distance range (20–300 m) showed an intermediate value. The mean decay rates for CO are always largest in distance range 20–100 m. But the mean decay rates in distance range 100–200 m are sometimes larger than the values for the whole distance range (20–300 m). The mean decay rates for NO_x_ and NO_2_ are always larger in distance range 20–100 m than in distance range 100–200 m while the whole distance range (20–300 m) showed intermediate values.

### 3.4 Meta-data analysis on decay rates

**Fig. 1:**
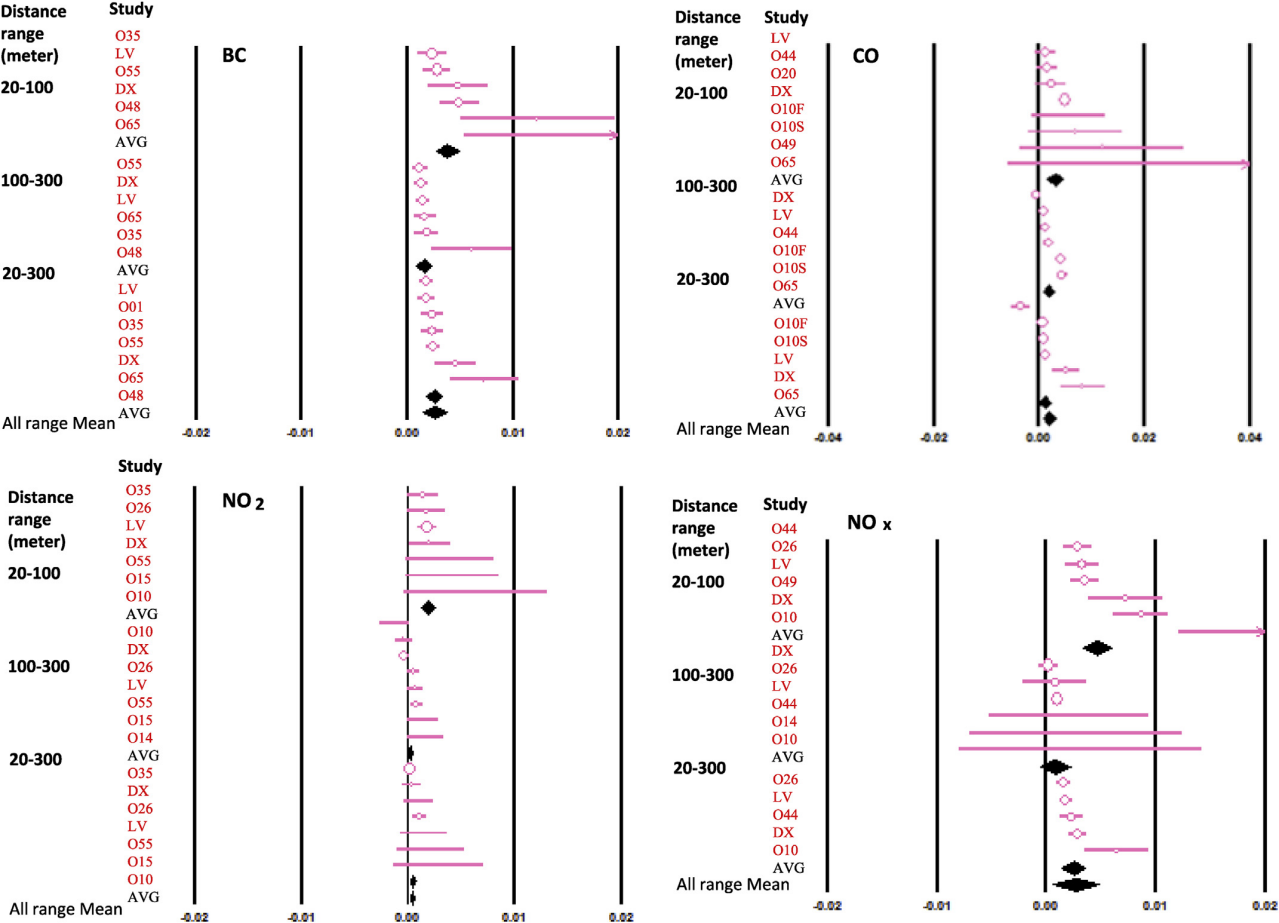
Meta-data analysis of decay rates for selected traffic-related air pollutants. The different types of air pollutants are shown in separate plots for Black carbon (BC), carbon monoxide (CO), nitrogen dioxide (NO_2_) and nitrogen oxides (NO_x_), respectively. A red line with open circle represents the 95% confidence intervals (CI) and the mean each individual study. Diamond under each set of distance range represents the average of a meta-analysis for that distance rage. The diamond aligned with “All range mean” at the bottom of each plot refers to meta-analysis on all three ranges for a given pollutant. Study IDs are as explained in Table 3.

Using a random model of meta-data analysis, we showed the means and their confident interval of air pollutant concentration decay rates among different studies and the overall from meta-analyses (Fig. 1). In general, smaller variations in the decay rates were seen in most studies on BC than the other air pollutants. The variation of average decay rates in individual studies were statistically significant for BC but not for other air pollutants (data not shown). However, the decay rate variations were significant on the meta-data analysis level for all air pollutants in the examination.

The meta-data analysis-based decay rates for BC are 0.0038, 0.0016, and 0.0025 m^−1^ for distance ranges 20–100, 100–300, and 20–300 m, respectively. The meta-data analysis-based decay rates for CO are 0.0030, 0.0019, and 0.0011 m^−1^ for distance ranges 20–100, 100–300, and 20–300 m, respectively. The meta-data analysis-based decay rates for NOx and NO_2_ are 0.0047 and 0.0019, 0.0008 m^−1^ and 0.0002, and 0.0025 and 0.0005 m^−1^ for distance ranges 20–100, 100–300, and 20–300 m, respectively.

The overall meta-data analysis-based decay rates are 0.0026, 0.0019, 0.0004, and 0.0027 m^−1^ for BC, CO, NO_2_ and NOx, respectively (Fig. 1).

### 3.5 Decay rate-based concentration prediction over distance from road

**Fig. 2:**
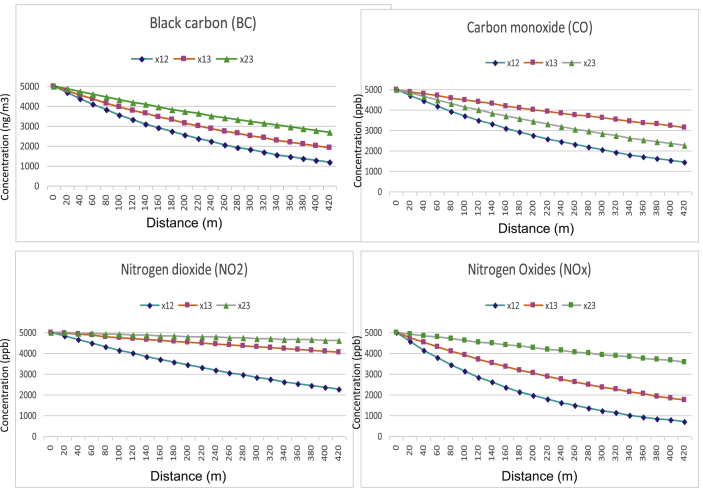
Near-road concentration predictions for selected traffic-related air pollutants. Legends x12, x13, and x23 inside each plot represents prediction based on meta-data analysis-derived decay rates for 20–100-m, 20–300-m, and 100–300-m, respectively.

Having obtained meta-data analysis-derived air pollutant decay rates based on real measurement data from multiple studies it is convenient and also useful to make some predictions on distance-concentration profiles for the selected air pollutants. We used an initial concentration of 5000 ug/m^3^ for BC or 5000 ppb for CO, NO_2_ and NOx and predicted their concentration decay patterns when different decay rates were used (Fig. 2).

Based on these predictions it is calculated that about 32% BC, 26% CO, 17% NO_2_, and 37% NOx would be decreased at 100 m from the road and about 59% BC, 58% CO, 21% NO_2_, and 52% NOx would be reduced at 300 m from the road.

### 3.6 Concentration prediction with confident interval among different decay rates

**Fig 3:**
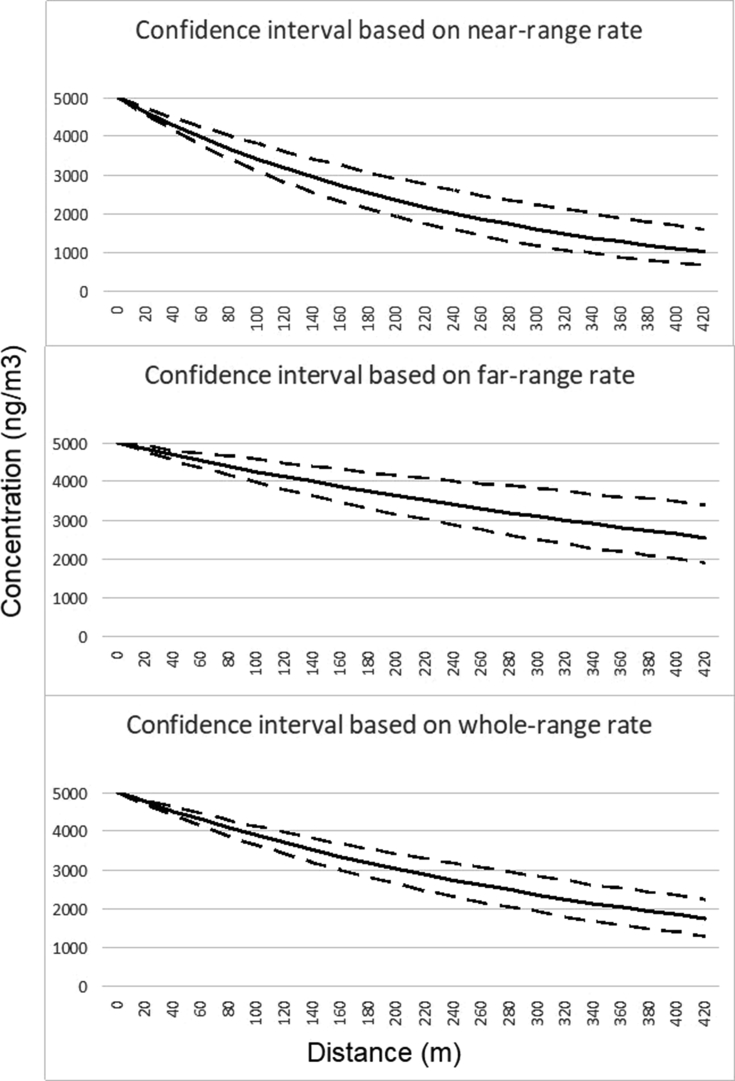
Confidence intervals of black carbon concentration predictions based on different decay rates. Meta-data analysis-derived decay rates for distance ranges 20–100-m, 20–300-m, and 100–300-m are used for making predictions shown at the top, middle, and bottom plots, respectively. The mean and the 95% upper and low limits are represented by solid and dashed lines, respectively.

The concentration decay of mobile-source air pollutants is faster in the near road distance range than the further away from road distance range. Thus, using different rates for concentration calculation will have some effect on the scope of the prediction. To illustrate this rate-selection effect on concentration prediction we present the concentration decay patterns and the 95% confidence intervals of BC (Fig. 3).

## 4 Discussion

Near-road air pollution is a very hot topic in environmental research which could be reflected with an easy retrieval of more than 6000 publications matching general terms on this topic. However, when we tried to find specific information on spatial extent or spatial patterns of traffic-related air pollutants near roads, we were disappointed to learn that many publications did not contain measurement data with desired spatial resolution. This limitation could reflect the difficulties in initiating near-road measurement studies which would be cost-expensive at least. In comparation with a previous study which showed 33 studies in the literature meeting its selection criteria for examining spatial extent of mobile source air pollution [26] and an important study which synthesized findings on near-roadway air quality collected data from 41 roadside monitoring studies in publications from 1978 to 2008 [40], our study was initially based on 65 publications with some measurement data (Table 1).

Further evaluation of these 65 publications indicated that PMs showed no apparent concentration decay over near-road distances, we focused our spatial profile analysis of concentration decay only on BC, CO, NO_2_ and NO_x._ We found 12 unique studies contained sufficient concentration and distance measurement information for our concentration decay analysis (Table 2).

A major problem in comparing different studies on near-road air pollutants is the widely varied initial on-road or road-edge concentrations in different studies. This variation greatly influences the spatial extent of the traffic emission-released air pollutants. A study [40] used two different normalization methods (background vs. edge normalization) to investigate the near-road air pollutant concentration decay. It was found that, for almost all pollutants, the influence of the roadway on air pollution concentrations decays to background was 115–570 m and 160–570 m according to the background normalization and the edge normalization, respectively. This study also found that the changes in pollutant concentrations with increasing distance from the road fell into one of three groups: rapid decay (e.g., CO), consistent decay (e.g., NO_2_) and no apparent decay (e.g., PM).

Thus, it appeared that using concentration decay rates may be a better way to describe “spatial dynamics” of air pollutants, regardless of methods of concentration normalization [41]. Pollutant concentration decay rates are often inclusive for many confounding factors and thus many variations such as local land use, season and time are absorbed into decay rates without a need to differentiate their compounding impacts. A previous modeling study of concentrations of nitrogenous compounds with distance from roads has led to a selection of exponential decay model for meaningful reflection of the measurement data [20]. Another study has found a logarithmic relationship between NO_2_ concentration and the distance from a highroad [21]. Some more complicated models have been used for characterizing air dispersion of CO and diesel particulate matter (DPM) [22].

In this study we used a simple exponential decay model to characterize the concentration decay of all selected air pollutants. Because many roads are built in such a way with clear buffering zone near the roads and more building structures are found with increasing distance from the roads, we calculated concentration decay rate in three different distance ranges as 20–100 m, 100–300 m and 20–300 m. We found that the fitting of this model to the spatial patterns of the traffic-related air pollutants was better if the concentration decay was calculated using different decay rates for the closer-to-road (20–100 m) and the further away-from-road (100–300 m) distance ranges, rather than using a single rate to cover the entire distance range (20–300 m). This phenomenon may be a reflection that traffic emission-released air pollutants in the near-road clear buffering zone (20–100 m) and more distance from the road (100–300 m) were influenced by different sets of factors which shaping their concentration dynamics.

Based on these model calculations it is predicted that about 32% and 59% of BC would decreased within 100- and 300-meters, respectively. About 26% and 58% of CO would disappear within 100- and 300-meters, respectively. About 17% and 21% of NO_2_ and about 37% and 52% of NOx would disappear within 100- and 300-meters, respectively. Such information would be valuable in assessing the exposure potential of these air pollutants once their emission rate and thus initial concentrations can be found or estimated.

In comparing the concentration decay patterns of different air pollutant in the selection we found that BC appeared to be the “best indicator” for near-road air pollution modeling because its much “tighter” dispersion trend in the meta-data analysis than those revealed for the other air pollutants such as CO, NO_2_ and NO_x_. CO showed a “reversed” decay rate for having a faster decay rate for the whole distance range than the far-way distance range. The reason for this anomaly in this study is unknown but it could be a reflection of limited data with extreme outliers.

We should point out that, by applying a very restricted set of selection criteria and limiting our analysis only on down-wind measurement data, the results of our meta-analysis would certainly not be an inclusive reflection of some different spatial patterns for near-road air pollutants that may exist in certain locations. The utilization of a simple exponential equation for calculating decay rate is a kind of arbitrary. Thus, provision of decay rates calculated for different distance ranges may be necessary and useful for exposure assessment and development planning in different urban communities.

## 5 Conclusion

With a focused literature search for near-road studies on air pollutants with concentration measurements and distance records we performed a concentration decay rate-based metaanalysis for major traffic-related air pollutants. In general, BC, CO, NO_2_ and NO_x_ followed the same pattern of logarithmic concentration decay. BC appeared to be an ideal candidate for near-road air pollution modeling because its consistent dispersion trend reflected in the meta-data analysis. This information are useful for enhancing exposure modeling studies on near-road traffic air pollutants and thus more reliably assessing the health risk of near road air pollution.

## Declarations

### Author contribution statement

Shi V. Liu, Fu-lin Chen, Jianping Xue: Conceived and designed the experiments; Performed the experiments; Analyzed and interpreted the data; Contributed reagents, materials, analysis tools or data; Wrote the paper.

### Funding statement

This research did not receive any specific grant from funding agencies in the public, commercial, or not-for-profit sectors.

### Competing interest statement

The authors declare no conflict of interest.

### Additional information

No additional information is available for this paper.
